# Derivation and characterization of an HIV-1 mutant that rescues IP_6_ binding deficiency

**DOI:** 10.1186/s12977-021-00571-3

**Published:** 2021-08-28

**Authors:** Daniel Poston, Trinity Zang, Paul Bieniasz

**Affiliations:** 1 Laboratory of Retrovirology, The Rockefeller University, New York, NY USA; 2 Weill Cornell/Rockefeller/Sloan-Kettering Tri-Institutional MD-PhD Program, New York, NY USA; 3 Howard Hughes Medical Institute, New York, NY USA

**Keywords:** HIV-1, IP_6_, Inositol phosphate, HIV-1 assembly, Imaging

## Abstract

**Background:**

A critical step in the HIV-1 replication cycle is the assembly of Gag proteins to form virions at the plasma membrane. Virion assembly and maturation are facilitated by the cellular polyanion inositol hexaphosphate (IP_6_), which is proposed to stabilize both the immature Gag lattice and the mature capsid lattice by binding to rings of primary amines at the center of Gag or capsid protein (CA) hexamers. The amino acids comprising these rings are critical for proper virion formation and their substitution results in assembly deficits or impaired infectiousness. To better understand the nature of the deficits that accompany IP_6_ binding deficiency, we passaged HIV-1 mutants that had substitutions in IP_6_ coordinating residues to select for compensatory mutations.

**Results:**

We found a mutation, a threonine to isoleucine substitution at position 371 (T371I) in Gag, that restored replication competence to an IP_6_-binding-deficient HIV-1 mutant. Notably, unlike wild-type HIV-1, the assembly and infectiousness of resulting virus was not impaired when IP_6_ biosynthetic enzymes were genetically ablated. Surprisingly, we also found that the maturation inhibitor Bevirimat (BVM) could restore the assembly and replication of an IP_6_-binding deficient mutant. Moreover, using BVM-dependent mutants we were able to image BVM-induced assembly of individual HIV-1 particles assembly in living cells.

**Conclusions:**

Overall these results suggest that IP_6_-Gag and Gag-Gag contacts are finely tuned to generate a Gag lattice of optimal stability, and that under certain conditions BVM can rescue IP_6_ deficiency. Additionally, our work identifies an inducible virion assembly system that can be utilized to visualize HIV-1 assembly events using live cell microscopy.

**Supplementary Information:**

The online version contains supplementary material available at 10.1186/s12977-021-00571-3.

## Background

The HIV-1 Gag polyprotein which is composed of the matrix (MA), capsid (CA), spacer peptide 1 (SP1), nucleocapsid (NC), spacer Peptide 2 (SP2), and p6 domains, has central structural and functional roles in the HIV-1 replication cycle. During virion assembly, multimerization of the Gag polyprotein at the plasma membrane, primarily driven by the CA and NC domains, generates immature HIV-1 virions composed of radially oriented Gag hexamers [1–5]. Following assembly, and concomitant with or shortly after nascent particles are released, proteolytic processing of Gag by HIV-1 protease separates the aforementioned Gag domains [6]. The liberated CA protein undergoes a major structural rearrangement to form the mature conical core, composed of a lattice of CA hexamers with 12 CA pentamers, and is the salient feature of particle maturation [7]. Only after maturation are HIV-1 particles able to initiate new cycles of infection.

It has been previously shown that inositol phosphates play a critical role in both HIV-1 assembly and maturation. While assembly of HIV-1 Gag protein in vitro yields immature particles that differ in size and character from authentic virions, addition of inositol phosphates to in vitro assembly reactions enables the production of particles that resemble authentic virions [8]. Further work identified inositol hexakisphosphate, or IP_6_, as the key mediator of this process. IP_6_, is a ubiquitous cellular polyanion containing 5 equatorial phosphates and a single axial phosphate, and facilitates formation of immature HIV-1 Gag lattice by binding to and stabilizing positively-charged rings of primary amines. These rings are formed by lysine residues at Gag positions 290 and 359 (K290 & K359) that are positioned at the center of the immature Gag hexamer [9]. Following the subsequent structural rearrangement of CA that accompanies maturation, IP_6_ next binds to a second, distinct positively charged ring in the mature CA hexamer formed by arginine residues at CA position 18 (R18, Gag position R150). The R18 ring stabilizes the mature CA hexamer, and is required for viral DNA synthesis in newly infected cells [10–12]. It is thought that IP_6_ is recruited into virions by interacting with K290 and K359 during immature particle production; this model is consistent with data demonstrating that HIV-1_K290A_ and HIV-1_K359A_ are significantly impaired in both viral production and IP_6_ packaging, while HIV-1_K359I_ is assembly competent but generates poorly infectious particles [13].

The importance of each of the IP_6_-coordinating residues has been established, as mutagenesis of any such residue to an alanine (HIV-1_R18A,_ HIV-1_K290A_, or HIV-1_K359A_) significantly impairs infectivity in either single cycle or spreading infection assays [9, 13]. Additionally, yield of infectious virions is also substantially reduced in cells lacking key enzymes in the IP_6_ biosynthetic pathway (IPPK or IPMK) or in cells overexpressing MINPP1, a phosphatase that dephosphorylates IP_6_ [9, 13–15]. The IP_6_-coordinating amino acids are conserved among diverse lentiviruses, suggesting a general requirement for IP_6_ [16].

While there is considerable evidence that perturbing IP_6_ binding impairs HIV-1 replication, further investigation into the precise mechanisms underlying replication deficits is warranted. To better understand the role of IP_6_, we serially passaged virions containing substitutions in IP_6_-coordinating residues to identify second-site compensatory mutations that might rescue the resulting infectivity deficits. Accordingly, we found a single substitution that rescued the replication deficit observed in two IP_6_ binding-deficient mutants. Using CRISPR/Cas9 knockout of IPMK, we show that the second-site substitution restored infectious virion yield despite loss of this IP_6_ biosynthetic pathway. Strikingly, we also found that treatment with a maturation inhibitor Bevirimat (BVM) rescues infectivity of the IP_6_-binding-deficient mutant HIV-1_K359A_. Indeed, using approaches in which the assembly of individual HIV-1 particles is imaged in living cells, we show that addition of BVM can induce the assembly of CA-mutant HIV-1 virions.

## Results

### ***Identification of a second-site substitution that restores replication competence to IP***_***6***_***-binding deficient HIV-1 mutants***

**Fig. 1:**
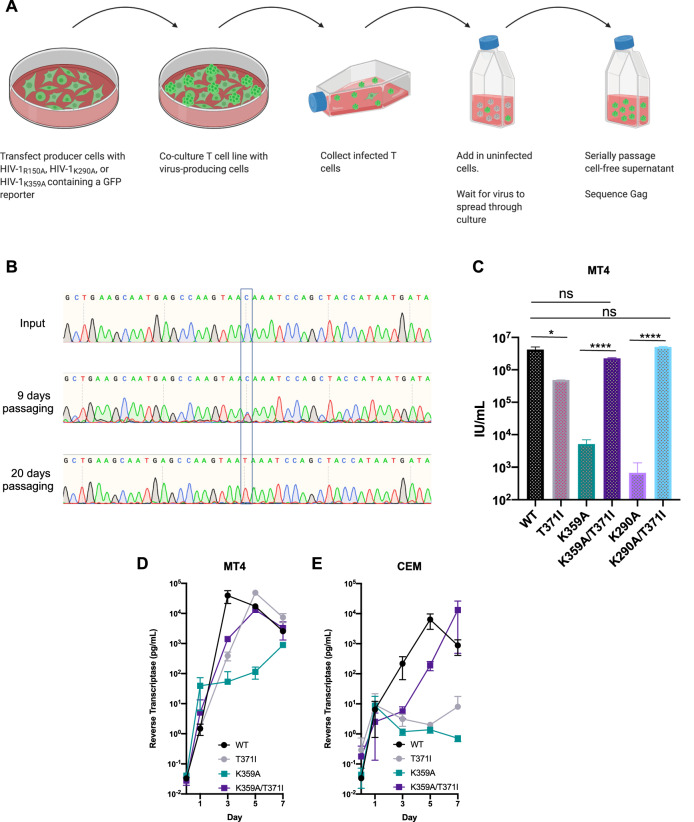
Derivation of an IP6-independent HIV-1 mutant. **A** Passaging schematic. **B** Sanger sequencing of HIV-1 gag indicating emergence of a single-site revertant. Input or viral RNA was isolated at indicated timepoint, and the gag region was amplified by RT-PCR. **C** Single cycle infection assay confirming the identified substitution rescues infectivity of K359A. 293T cells were transfected with proviral clones of WT HIV-1_NHG_ or indicated mutant, and 48 h post transfection, supernatant was titrated on MT4 cells. Statistical analysis: Student’s T test. **D**, **E** Spreading replication assays in MT4 cells (**D**) or CEM cells (**E**). Cells were infected with HIV-1_WT_ or indicated mutant at an MOI of 0.001. 16 h post infection, inoculum was washed away and supernatants were collected at the indicated timepoints. Reverse transcriptase activity was measured in the supernatant samples using SYBR-PERT

To identify second site changes that would rescue IP_6_ binding deficient mutants, we passaged HIV-1 mutants encoding substitutions in IP_6_ coordinating residues (HIV-1_R18A_, HIV-1_K290A,_ or HIV-1_K359A_) in the highly-permissive MT4 T-cell line. Initial attempts, in which MT4 cells were infected with mutant viral stocks, were unsuccessful, likely due to the dramatically impaired fitness of these mutants and consequent inability to establish a sufficiently large population of infected cells to generate revertants. To overcome this problem, we instead co-cultured MT4 cells with virus-producing 293T cells that had been transfected with HIV-1_R18A_, HIV-1_K290A,_ and HIV-1_K359A_ proviral plasmids that encode GFP in place of nef. After removing the 293T cells, infected MT4 cells were co-cultured with uninfected MT4 cells, until most of the MT4 cells became infected (as monitored by visual inspection of GFP+ cells in the culture). Thereafter, cell-free supernatant was serially passed in MT4 cells (Fig. 1A).

For one mutant, HIV-1_K359A_, observation of GFP positive cells suggested that an apparently compensatory mutation arose after approximately 2 weeks of passaging. PCR amplification and sequencing of Gag encoding sequences from this culture revealed the presence of a single nucleotide substitution in *gag* that resulted in a threonine to isoleucine substitution at Gag position 371 (Fig. 1B). No revertant mutants could be obtained for HIV-1_R18A_ or HIV-1_K290A_. This finding may reflect a greater magnitude of impairment of these particular substitutions, making the generation of revertant mutants more difficult.

To determine whether the T371I mutant rescued the infectivity defect present in HIV-1_K359A_, we generated a proviral clone, HIV-1_K359A/T371I_, encoding both mutations and measured the infectious virion yield from proviral plasmid-transfected 293T cells. Addition of the T371I substitution to HIV-1_K359A_ restored infectious virion yield to wild-type levels (Fig. 1C). Although this second-site, apparently compensatory change was identified only in the context of HIV-1_K359A_, we asked whether the T371I substitution could rescue the HIV-1_K290A_, given purported similar roles of K290 and K359 in binding IP_6_. Indeed, we found that HIV-1_K290A/T371I_, unlike HIV-1_K290A_, yielded similar levels of infectious HIV-1 virions to wild-type HIV-1.

To determine whether the effects of the T371I mutant were evident outside the context of transfected 293T cells, we performed spreading replication assays of HIV-1_WT_, HIV-1_T371I_, HIV-1_K359A_, and HIV-1_K359A/T371I_ in MT4 cells (Fig. 1D) and CEM cells (Fig. 1E). As expected, HIV-1_K359A_ replicated poorly in both cell types. HIV-1_T371I_ replicated poorly in CEM cells but well in MT4 cells, perhaps reflecting the greater permissiveness of MT4 cells. Importantly however, we found similar phenotypes for HIV-1_K359A/T371I_ in spreading replication assays in both MT4 and CEM cells; namely, addition of the T371I substitution to HIV-1_K359A_ restored replication, with the HIV-1_K359A/T371I_ double mutant exhibiting only a modest delay compared to HIV-1_WT_ in both cell types (Fig. 1D, E).

### ***Infectious HIV-1***_***K359A/T371I***_*** particle yield is not affected by reduction of IP***_***6***_*** synthesis in virus producing cells***

**Fig. 2:**
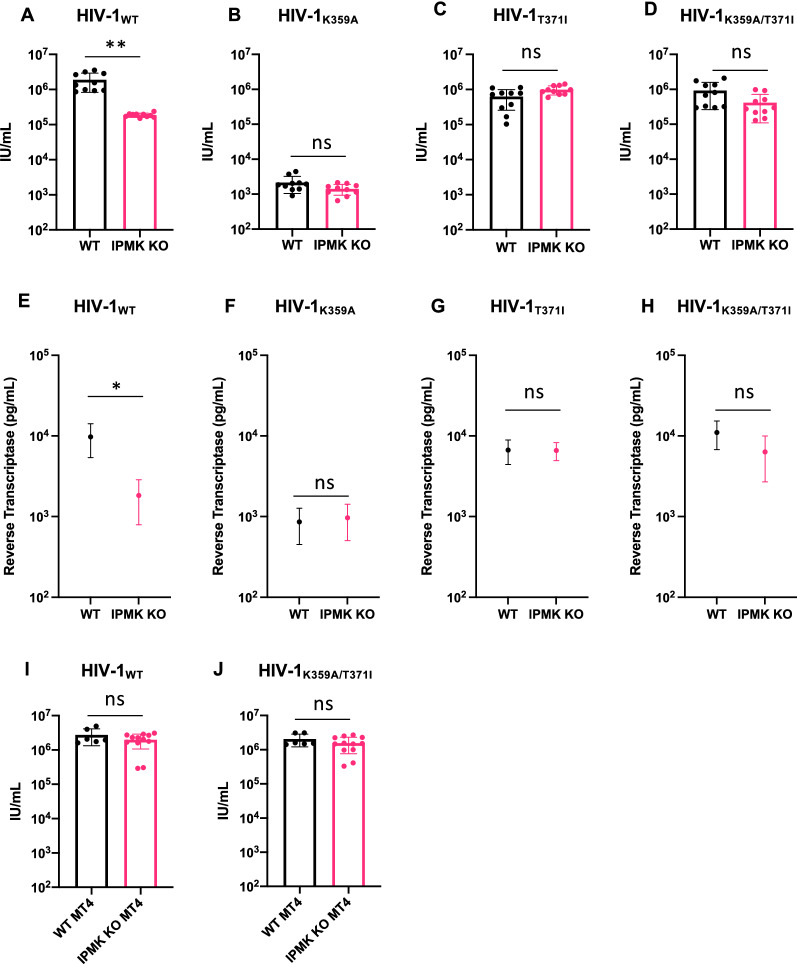
HIV-1_K359A/T371I_ is not impaired by lack of cellular IP6. **A**–**D** Control or IPMK KO single 293T cell clones were transfected with HIV-1_WT_ (**A**), HIV-1_K359A_ (**B**), HIV-1_T371I_ (**C**), or HIV-1_K359A/T371I_ (**D**) proviral plasmids. After 48 h, supernatants were collected and titrated on MT4 cells. Each data point represent a different 293T cell clone. Statistical analysis: unpaired Student’s t-test. Alternatively, levels of Reverse Transcriptase in supernatants were quantified using the SYBR-PERT assay (**E**–**H**). **I**, **J** HIV-1_WT_ (**I**) or HIV-1_K359A/T371I_ (**J**) virions were titrated on WT MT4 or IPMK KO MT4 cells and infection quantified by flow cytometry. Each data point represent a different MT4 cell clone. Statistical analysis: unpaired Student’s t-test

Because the HIV-1_K359A_ is defective for IP_6_ binding we next asked whether HIV-1_K359A/T371I_ retained infectiousness when cellular IP_6_ levels were reduced. Using CRISPR/Cas9 we generated 293T cell lines lacking IPMK, an enzyme in the IP_6_ synthetic pathway. Previous work has demonstrated IPMK knockout cells have greatly reduced levels of both IP_5_ and IP_6_ [13]. To account for potential clonal variation in capacity to generate HIV-1 particles, we used 3 separate IPMK targeting sgRNAs or a corresponding empty vector to generate ten independent single cell clones of IPMK knockout and WT control 293T cells (Fig. 2A). The loss of IPMK was confirmed by DNA sequencing of target loci, which revealed the introduction of frameshift mutations and the absence of intact IPMK alleles. In agreement with previous studies, the yield of infectious HIV-1_WT_ virions from IPMK-deficient 293T cells was significantly decreased, by tenfold (p = 0.0091, Fig. 2A). The yield of HIV-1_K359A_ from 293T cells was greatly reduced compared to wildtype HIV-1 as expected, and was not further reduced by IPMK deficiency (Fig. 2B). Yield of HIV-1_T371I_ was also slightly reduced compared to wildtype but not impacted by IPMK deficiency (Fig. 2C, p = 0.1374). Importantly, the yield of HIV-1_K359A/T371I_ was only marginally reduced compared to wild type HIV-1 and there was no difference in yield of infectious HIV-1_K359A/T371I_ from WT 293T cells versus IPMK deficient 293T cells (Fig. 2D, p = 0.178).

As IP_6_ deficiency impacts both HIV-1 particle production as well as infectivity, we assayed particle levels in the same supernatants from Fig. 2A–D using detection of reverse transcriptase with the SYBR-PERT assay. Importantly, we see the same phenotype for particle production as we do with infectivity assays: production of HIV-1_WT_ particles is impaired in IPMK KO cells, but there are no production deficits for HIV-1_K359A_, HIV-1_T371I_, or HIV-1_K359A/T371I_ (Fig. 2E–H). However, while there was a tenfold reduction in HIV-1_WT_ infectivity from IPMK-deficient 293T cells, we only observed a fivefold reduction in particle production in the exact same supernatants.

### ***Infection of target cells with impaired IP***_***6***_*** synthesis by HIV-1***_***WT***_*** or HIV-1***_***K359A/T371I***_

It has been proposed that residues K290 and K359 recruit IP_6_ into HIV-1 virions during assembly, thereby providing the source of the IP_6_ that binds to and stabilizes the R18 ring in the mature capsid core. The rationale for this idea stems from previous studies which have demonstrated that reduction of cellular IP_6_ levels in target cells does not impact susceptibility to incoming infection [13, 14]. Because HIV-1_K359A/T371I_ is fully infectious despite encoding a mutation that is predicted to diminish IP_6_ packaging into virions, we next asked whether HIV-1_K359A/T371I_ requires IP_6_ in target cells to be maximally infectious. We generated twelve IPMK-deficient MT4 target cell clones and six control clones and performed single cycle infection assays using HIV-1_WT_ and HIV-1_K359A/T371I_ (Fig. 2D, E). In agreement with previous studies [13], there was no difference in the infectiousness of HIV-1_WT_ in WT or IPMK-deficient MT4 cells (Fig. 2D p = 0.3863). Moreover, there was no deficit in the infectiousness of HIV-1_K359A/T371I_ in WT or IPMK-deficient MT4 target cells (Fig. 2E, p = 0.4331), suggesting that HIV-1_K359A/T371I_ either does not require IP_6_ for replication, or that the T371I mutation rescues both replication and IP_6_ incorporation.

### ***Bevirimat rescues infectious virion formation by the IP***_***6***_***-binding deficient mutant HIV-1***_***K359A***_

**Fig. 3:**
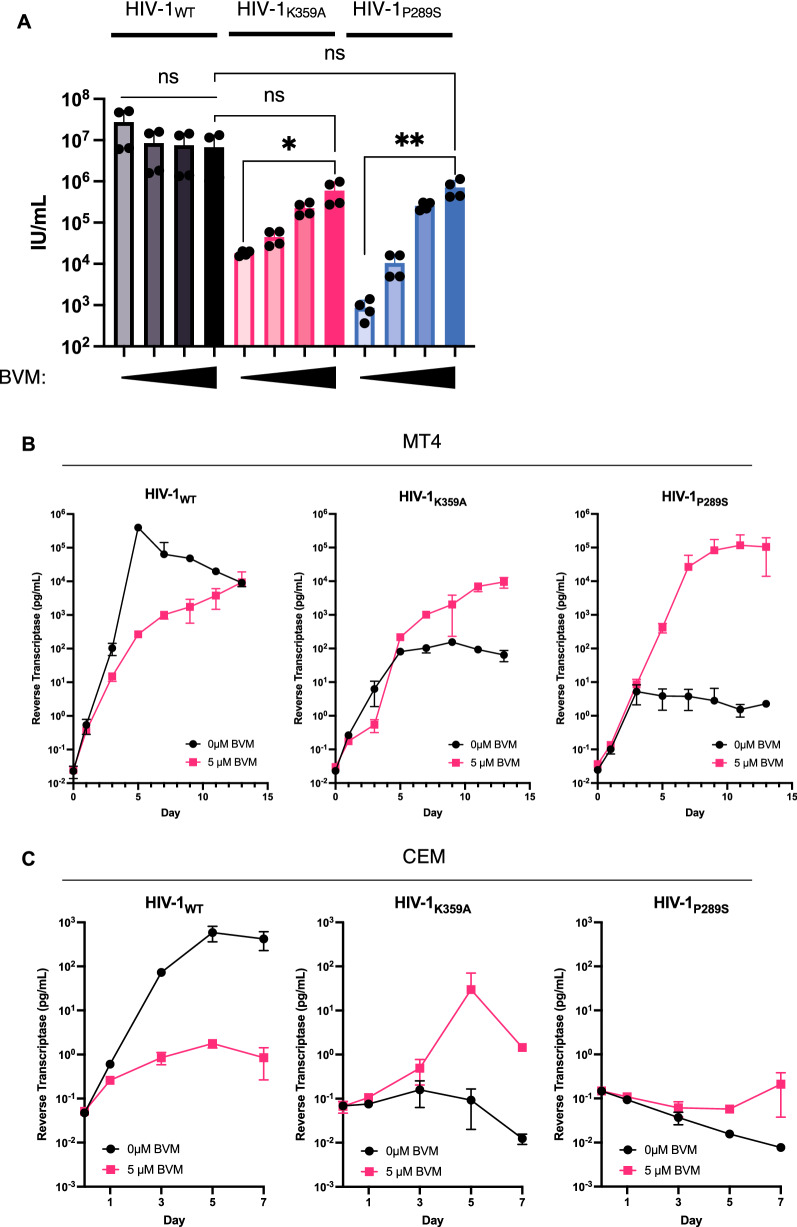
Bevirimat rescues infectivity of HIV-1_K359A_. **A** 293T cells were transfected with HIV-1_WT_, HIV-1_K359A_, or HIV-1_P289S_ proviral plasmids in the presence of 0, 1, 5, or 10 μM Bevirimat. After 48 h, supernatants were collected and titrated on MT4 cells and infection quantified by flow cytometry. **B**, **C** MT4 cells (**B**) or CEM cells (**C**) were infected with HIV-1_WT_, HIV-1_K359A_, or HIV-1_P289S_ at an MOI of 0.001. At 16 h post infection, inoculum was washed away and supernatants were collected at the indicated timepoints. Reverse transcriptase activity was measured in the supernatant samples using SYBR-PERT

Notably, The T371I mutation identified herein had been described previously in a different context. Specifically, this substitution was reported to stabilize the immature CA-SP1 lattice, mimicking the effect of maturation inhibitors (MI) [17, 18]. Therefore, we next asked whether maturation inhibitors themselves could rescue the deficit in infectious virion yield exhibited by HIV-1_K359A_. As a control, we included the previously described assembly-defective, maturation inhibitor-dependent CA mutant HIV-1_P289S_ [18], We found that that BVM indeed rescued the infectivity of HIV-1_K359A_ and HIV-1_P289S_ in both single-cycle and spreading replication. Specifically, in single cycle assays, BVM increased the yield of infectious HIV-1_K359A_ virions, up to 50-fold, and in a dose-dependent manner (Fig. 3A) from transfected 293T cells. In spreading replication assays, BVM restored HIV-1_K359A_ replication to levels similar to that of BVM-treated wildtype virus in MT4 cells (Fig. 3B). BVM also rescued the spreading replication of HIV-1_K359A_ in CEM cells, indeed in this context the effect of BVM on HIV-1_K359A_ spreading was greater than that on the previously described MI-dependent mutant HIV-1_P289S_ (Fig. 3C).

### ***BVM increases release of HIV-1***_***K359A***_*** virions independently of the viral protease***

**Fig. 4:**
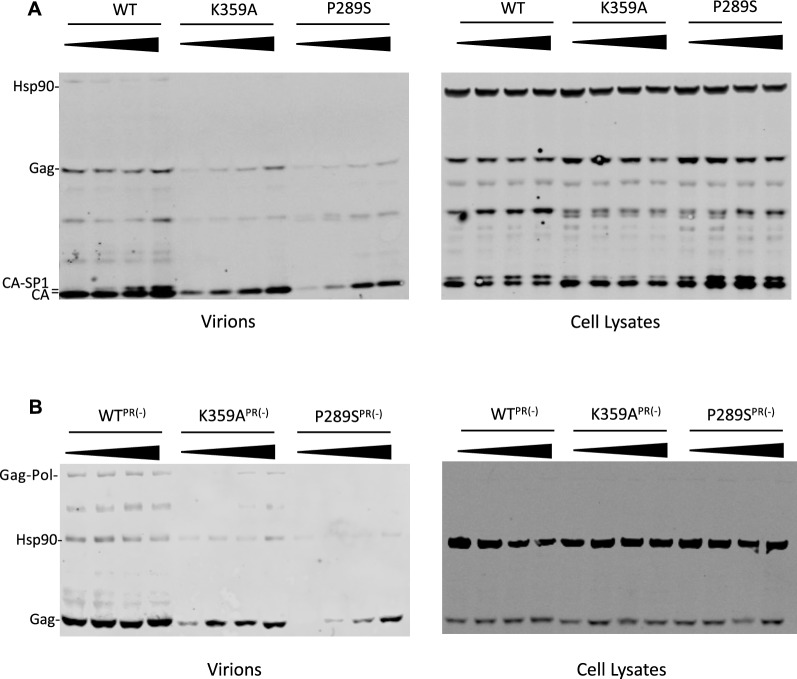
Bevirimat increase release of HIV-1_K359A_ independently of protease inhibition. **A** 293T cells were transfected with HIV-1_WT,_ HIV-1_K359A,_ or HIV-1_P289S_ in the presence of 0, 1, 5, or 10 μM Bevirimat. 48 h post transfection, supernatants were pelleted over a sucrose cushion and analyzed by SDS-PAGE alongside cell lysates. Hsp90 serves as a loading control. **B** 293T cells were transfected with HIV-1_WT_, HIV-1_K359A,_ or HIV-1_P289S_ bearing an inactivating mutation in protease in the presence of 0, 1, 5, or 10 μM Bevirimat and analyzed via SDS-PAGE as above. Hsp90 serves as a loading control

The interaction between K359 amines and IP_6_ likely stabilizes the immature Gag lattice, Similarly, maturation inhibitors are known to bind to the immature Gag hexamers at approximal site and stabilize the immature CA-SP1 lattice [19, 20]. Therefore, we hypothesized that BVM rescue particle formation by HIV-1_K359A_ by stabilizing an otherwise destabilized lattice, effectively serving as a functional replacement for IP_6_. To test this idea, we measured the release of HIV-1_K359A_ virions from BVM-treated 293T cells by western blotting. BVM indeed increased the yield of HIV-1_K359A_ virions, in a dose-dependent manner (Fig. 4A). To confirm that this effect was not due to BVM-mediated inhibition of Gag proteolysis, we performed similar experiments in virions containing an inactivating mutation in protease. We observed a similar dose-dependent increase in immature particle release, even in the context of protease inactivation (Fig. 4B), suggesting that the effect of BVM on HIV-1_K359A_ assembly is due to the direct effect of BVM on the immature lattice, not through inhibition of proteolytic cleavage at the Gag-SP1 junction.

### Visualization of BVM-induced HIV-1 assembly observed in real time using live cell fluorescence microscopy

The above data strongly suggested that BVM rescues infectivity and release of HIV-1_K359A_ by facilitating particle assembly. To directly observe effects on virion assembly, we performed fluorescence microscopy using a novel imaging construct based on HIV-1_NL4-3,_ in which Pol has been replaced by an HIV-1 codon-mimicking mNeonGreen, and in which Env and Vpu bear inactivating mutations. The resulting construct, herein referred to as HIV-1 NG, generates Gag-mNeonGreen in place of Gag-Pol during a single cycle of infection, thus allowing visualization of particle assembly as punctae at the plasma membrane.

**Fig. 5:**
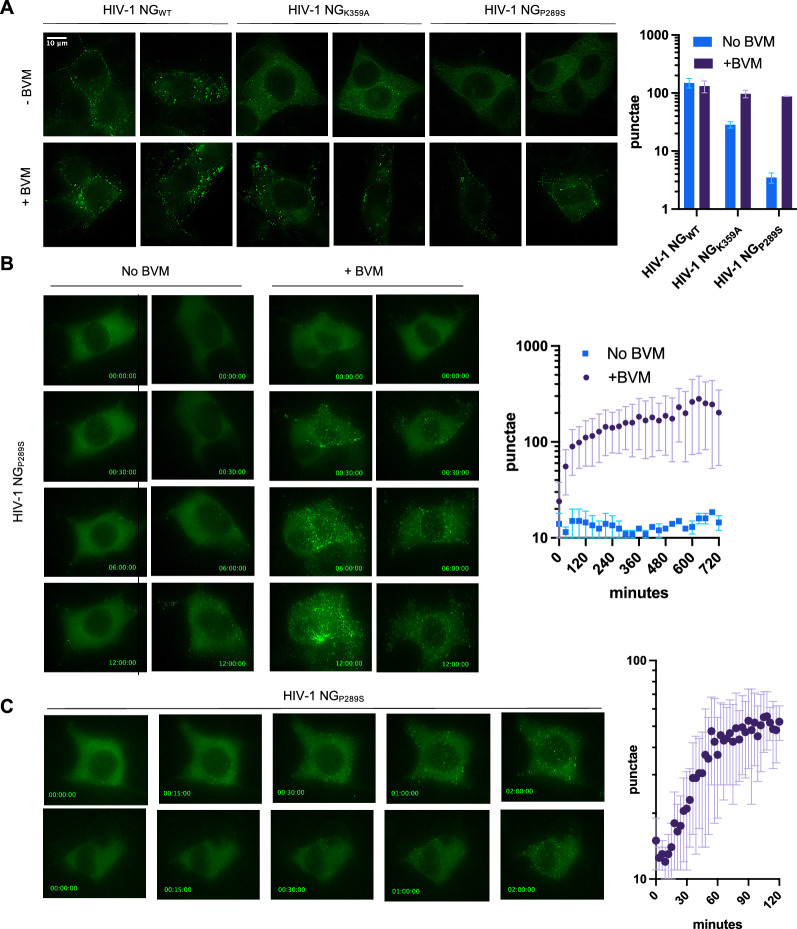
Visualization of Bevirimat-induced assembly by fluorescence microscopy. **A** Fluorescence microscopy of TZM cells infected with a reporter HIV-1 virus encoding mNeon Green in place of Pol (HIV-1 NG) to visualize assembly. Representative images 48 h-post-infection of HIV-1 NG_WT_, HIV-1 NG_K359A_, or HIV-1 NG_P289S_ infected in the absence or presence of 5 μM BVM. Quantification of Gag-NG punctae per image depicts mean ± SD for images from (A). **B** Representative time lapse images of TZM cells infected with HIV-1 NG_P289S_ −/+ treatment with 5 μM BVM at 26 h post infection. Image acquisition began immediately after BVM addition. Image labels: Hours:Minutes:Seconds post BVM addition. Quantification of Gag-NG punctae per timepoint depicts mean ± SD for images from **B**. **C** Representative time-lapse images of TZM cells treated with BVM at 26 h post infection with HIV-1 NG_P289S._ Image labels: Hours:Minutes:Seconds post BVM addition. Quantification of Gag-NG punctae per timepoint depicts mean ± SD for images from **C**

We generated HIV-1 vectors particle containing this reporter and derivatives (HIV-1 NG_WT,_ HIV-1 NG_K359A_, and HIV-1 NG_P289S_) by supplying Gag-Pol and VSV-G *in trans.* Then, we infected TZM-bl cells in the absence or presence of 5 μM BVM and performed widefield imaging on fixed cells 48 h post infection. In the absence of BVM, cells infected with HIV-1 NG_K359A_ and HIV-1 NG_P289S_ exhibited primarily diffuse cytoplasmic fluorescence, and fewer punctae than for HIV-1 NG_WT_ infected cells, indicating impaired assembly (Fig. 5A). However, when infections were done in the presence of BVM, there were clearly increased numbers of membrane associated punctae in HIV-1 NG_K359A_ and HIV-1 NG_P289S_ infected cells (Fig. 5A) suggesting BVM is able to directly facilitate particle assembly by these mutants.

This ability to induce HIV-1 particle assembly via addition of an exogenous small molecule has potential applications in imaging and other studies, as an inducible particle assembly system. In order to test the possible utility of this approach, we performed live cell widefield imaging studies using HIV-1 NG_P289S_, as this mutant displayed a greater responsiveness to BVM-induced assembly (see Figs. 3A, B, 4B, and 5A). We infected TZM-bl cells in the absence of BVM and then, at 26 h after infection, added BVM 5 μM and began acquiring images at 30 min intervals. In the absence of BVM, few punctae are apparent in HIV-1 NG_P289S_ infected cells, even after 12 h of imaging (Fig. 5B, Additional files 1, 2). However, in the BVM-treated cells, substantially more punctae were evident, as soon as 30 min after BVM addition (Fig. 5B, Additional files 3, 4).

**Fig. 6:**
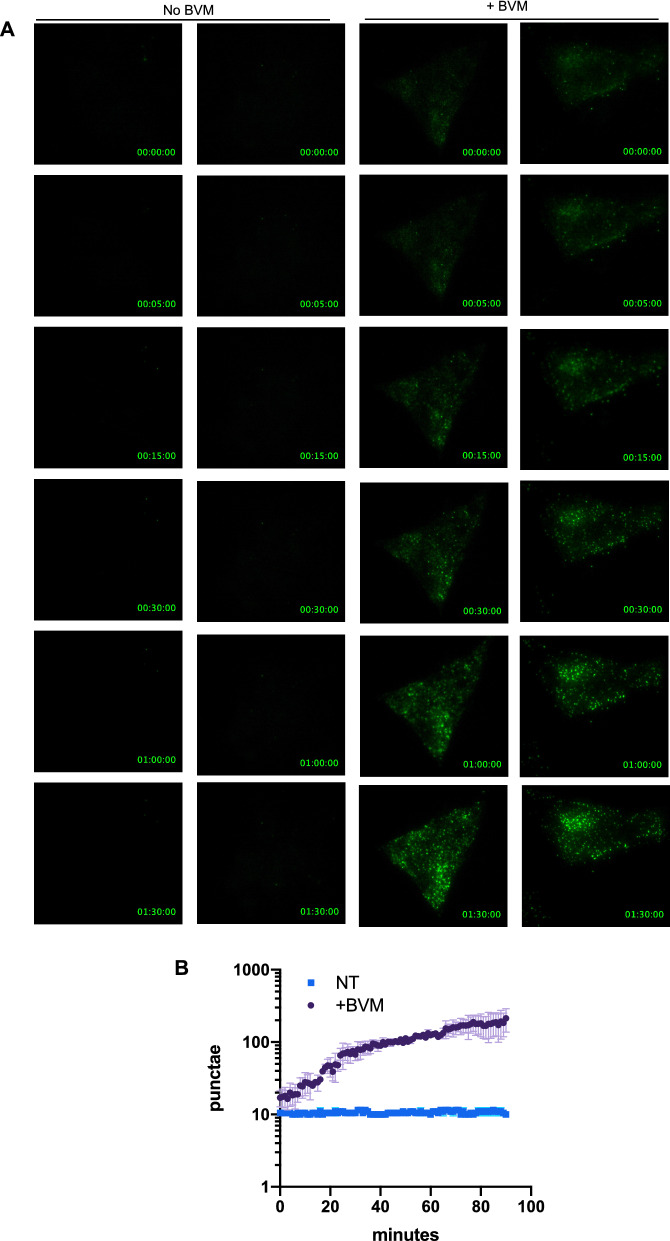
Visualization of Bevirimat-Induced assembly by TIR-FM. **A** TIR-FM microscopy of TZM cells infected with HIV-1_P289S_ NG. Representative time-lapse images of TZM cells treated with BVM at 28 h post infection_._ Image labels: Hours:Minutes:Seconds post BVM addition. **B** Quantification of punctae per time point depicts mean ± SD for images from **A**

Given that such striking differences could be observed as early as 30 min post BVM addition, we repeated these experiments with increased time resolution. TZM-bl cells were infected with HIV-1 NG_P289S_ for 26 h and treated with BVM as above, followed by immediate image acquisition at 3 min intervals. Assembly of HIV-1_P289S_ was rapidly induced by BVM (Fig. 5C, Additional files 5, 6) with substantial numbers of punctae forming within 30–120 min. We performed similar experiments using TIR-FM imaging and quantified the presence of Gag-NG punctae over time, with similar results. Specifically, in the absence of BVM there were very few punctae evident at the plasma membrane (Fig. 6A, B, Additional files 7, 8). However, shortly after the addition of BVM, numerous punctae rapidly formed at the plasma membrane (Fig. 6, Additional files 9, 10). These data provide proof of principle that such a system could be used to experimentally manipulate HIV-1 assembly for imaging or functional studies.

## Discussion

Here, we report the identification of a single amino acid substitution (T371I) that rescues the replication of the defective, IP_6_-binding deficient mutant HIV-1_K359A_. Despite several attempts we were unable to generate revertant mutants for HIV-1_K290A_ and HIV-1_R18A_ (although follow-up studies demonstrated that the T371I substitution rescues HIV-1_K290A_ as well as HIV-1_K359A_). The inability to generate revertant mutants for HIV-1_R18A_ and HIV-1_K290A_ is likely due to more substantial impairment. Indeed, previous groups have shown that HIV-1_K290A_ is impaired to a greater extent than HIV-1_K359A_, potentially because K290 binds the 5 equatorial phosphates on IP_6_ while K359 coordinates the single axial phosphate, suggesting a greater role for K290 in coordinating IP_6_ [13]. The inability to generate a revertant mutant rescuing HIV-1_R18A_ could be explained by functions of this residue in addition to coordinating IP_6_, such as recruitment the cellular protein FEZ1 or as service as a conduit for dNTPs into the mature core [10, 11, 21, 22].

HIV-1_K359A/T371I_ was fully infectious despite containing a mutation (K359A) that renders VLP assembly unresponsive to IP_6_ in vitro and which substantially impairs IP_6_ incorporation into virions [9, 13]. Indeed, we found no significant reduction in yield of HIV-1_K359A/T371I_ from IPMK KO 293T cells, in contrast to WT HIV-1. This finding suggests that HIV-1_K359A/T371I_ is no longer dependent on IP_6_ or requires substantially lower concentrations of IP_6_ in virus producing cells.

In addition to its role in promoting immature particle assembly, IP_6_ has also been implicated in stabilizing the mature lattice and promoting viral DNA synthesis by binding to a positively charged pore formed by R18 residues in the mature CA lattice [10, 21]. Previously it was proposed that the source of the IP_6_ required to stabilize the mature lattice in virions is selective recruitment by K290 and K359 residues, with IP_6_ being liberated to bind R18 residues following disruption of the immature lattice after proteolysis [9, 10]. In agreement with previous studies, we found that IPMK KO target MT4 cells were fully susceptible to infection by HIV-1_WT_, indicating that IP_6_ from target cells is not required to initiate a productive cycle of infection [13]. However, we also found no difference in the infectiousness of HIV-1_K359A/T371I_ in WT and IPMK KO MT4 target cells. This may reflect the possibility that other polyanions can fulfil a post assembly role. Indeed, recent studies demonstrated that other polyanions in mammalian cells such as glucose-1,6-bisphosphate can stabilize mature HIV-1 cores in vitro [23]*.* Alternatively, a very recent report has demonstrated that addition of the T371I substitution can rescue IP_6_ incorporation in HIV-1_K359A_ virions to near WT levels [24]. Nevertheless, our data utilizing genetic ablation of the IP_6_ synthetic machinery suggests that the T371I substitution confers reduced dependence on cellular inositol phosphate levels for virion assembly. Indeed, while production of HIV-1_WT_ was impaired in IPMK KO cells, there was no impairment of HIV-1_T371I_ or HIV-1_K359A/T371I_ in these cells. Thus HIV-1_T371I_ and HIV-1_K359A/T371I_ retains infectiousness even in the setting of reduced cellular IP_6_ levels, implying at least some level of IP_6_ independence, even if IP_6_ is incorporated into virions in the context of the HIV-1_K359A/T371I_ double mutant. Alternatively, the lack of impairment of HIV-1_T371I_ or HIV-1_K359A/T371I_ in the setting of reduced cellular IP_6_ levels maybe be due to the T371I substitution itself increasing affinity of the lattice for IP_6_, thus rendering virions less responsive to a reduction in cellular IP_6_ levels. This notion is consistent with the recent report by Mallery et al. [24], demonstrating that the T371I substitution rescues incorporation of IP_6_ in HIV-1_K359A._

The T371I substitution, identified herein as a compensatory mutation that rescues infectivity deficit found in HIV-1_K359A_, has previously been reported to rescue the infectivity of virions containing substitutions that confer resistance to maturation inhibitors (MIs). These substitutions render HIV-1 assembly-defective in the absence of MIs [18] and the T371I substitution was shown to stabilize the CA-SP1 lattice in this context, effectively mimicking the action of MIs [17]. Because the T371I substitution apparently mimics the effect of MIs, we hypothesized that MIs might also rescue the infectivity of HIV-1_K359A_. Strikingly, we found that this was the case, and found that BVM can stimulate the assembly and release of HIV-1_K359A_. That the stabilizing effects of both the T371I substitution and BVM can compensate for the lack of IP_6_ coordination in HIV-1_K359A_ provides in vivo mechanistic support for the model proposed by Dick et al.: i.e. that binding of IP_6_ to K290 and K359 residues stabilizes the immature lattice to drive particle assembly. Interestingly, while Mallery et al. [24] also demonstrated that MIs rescued HIV-1_K359A_, they observed that PF-46396, but not BVM, rescued infection. This is potentially due to differences in the concentration of BVM used (5.0 µM vs 0.5 µM).

The ability to promote assembly via addition of a small molecule has potential utility in imaging and other studies of HIV-1 assembly. Indeed, when performing live cell time-lapse imaging, we observed BVM-induced assembly on the timescale of minutes. Such an experimental approach has potential utility for studying the sequence of events in HIV-1 viron assembly, such are RNA packaging, ESCRT protein recruitment, and protease activation, where virion assembly can be rapidly and (potentially) reversibly induced in real time simply by addition of a small molecule.

Together, these data support a model whereby stability of the immature CA lattice is finely tuned, with IP_6_ coordinating and stabilizing the otherwise repulsive positive charges of K290 and K359 to drive assembly. Manipulations that cause IP_6_ binding deficiency, either mutagenesis of K359 or decreasing IP_6_ levels in producer cells, destabilize the immature lattice and decrease production of progeny virions. Conversely, manipulations such as the T371I substitution or treatment of HIV-1_WT_ with BVM, hyper-stabilize the immature lattice in the wild type context and decrease HIV-1 infectivity. However, either the T371I substitution or BVM treatment are able to rescue virion assembly and infectiousness in the context of IP_6_ deficiency. Understanding how small molecules such as BVM or IP_6_ can enhance Gag lattice stability can provide new tools to study virion assembly and potential avenues for antiretroviral therapeutics.

## Conclusion

We identified a single-site revertant mutation, T371I, that rescues replication competence of the IP_6_-binding-deficient mutant HIV-1_K359A_. Using CRISPR/Cas9 to genetically ablate IP_6_ biosynthesis, we showed that these resulting HIV-1_K359A/T371I_ virions are less dependent on cellular inositol phosphate levels. Remarkably, we also found that the maturation inhibitor BVM could restore the assembly and replication of HIV-1_K359A_ and developed an inducible particle assembly system using BVM-dependent HIV-1 mutants. In addition to providing insight on Gag-Gag and Gag-IP_6_ interactions during HIV-1 assembly, our work also identifies an inducible virion assembly system that can be used in investigating HIV-1 assembly events in living cells.

## Materials and methods

### Cells and media

293T cells were maintained in Dulbecco’s modified Eagle’s medium (DMEM, Gibco) supplemented with 10% fetal calf serum and gentamycin. MT4 cells were maintained in Roswell Park Memorial Institute (RPMI) 1640 Medium (Gibco) supplemented with 10% fetal calf serum and gentamycin. Cells were maintained at 37 °C and 5% CO_2_. All transfections with viral plasmids were performed with polyethyleneimine.

### Plasmid construction

All full-length proviral plasmids used in this study were based on the HIV-1 clone NHG, a previously described HIV-1 clone that encodes GFP in place of Nef (Accession number: JQ585717) [25]. Mutant viruses were derived from this parental plasmid using primer mutagenesis with fragments assembled into NHG digested with SpeI and SbfI using NEB HiFi DNA Assembly Master Mix according to the manufacturer’s instructions. Primers used for mutagenesis include: K359A F: 5ʹ-CCGGCCATGCTGCAAGAGTTTTG; K359A R: 5ʹ-CAAAACTCTTGCAGCATGGCCGG; T371I F: 5ʹ-GCAATGAGCCAAGTAATAAATCCAGCTACC; T371I R: 5ʹ-GGTAGCTGGATTTATTACTTGGCTCATTGC; P289S F: 5ʹ-CATAAGACAAGGAAGTAAGGAACCCTTTAGAG; P289S R: 5ʹ-CTCTAAAGGGTTCCTTACTTCCTTGTCTTATG; R57G F: 5ʹ-TCAAAGTAGGACAGTATGATC; R57G R: 5ʹ-GATCATACTGTCCTACTTTGA. The imaging construct HIV-1 NG was derived from NL4-3. The region of NL4-3 encoding Pol was deleted from bp 2294–4813 and a unique XbaI was added at bp 2301; Vpu was deleted and Env was truncated by removing bp 6056–7250 inserting NheI at 6056. These deletions and restriction sites were created through overlap PCR and cloned into NL4-3 via SphI and NheI (NL4-3 BssHII 5ʹ-GCTGAAGCGCGCACGGCAAGAGGCG 5ʹ-CTGAAGCGCGCACGGCAAGAGGCGAGG and dPol Xba AS 5ʹ-CTACTATTCTTTCCCCTGCACTCTAGACTACTACTTTATTGTGACGAGGGGTCGC; dPol Xba S 5ʹ-GCGACCCCTCGTCACAATAAAGTAGTAGTCTAGAGTGCAGGGGAAAGAATAGTAG and dVpudEnv NheI AS 5ʹ-CTCCTCGCTAGCGTACTACTTACTGCTTTGATAG). The sequence encoding neon green (NG) was codon optimized to have nucleotide composition and codon usage similar to that of Pol using the Codon Optimization On-Line Tool from Singapore University (http://cool.syncti/org) and was synthesized by GeneArt. Neon Green was fused into the p6* frame of Pol through overlap PCR and inserted via SphI and XbaI (NL4-3 SphI 5ʹ AGTGCATGCAGGGCCTATTGCACC, Pol-NG AS 5ʹ-CATGTTATCCTCCTCGCCCTTGCTCACCATCTTTATTGTGACGAGGGGTCGCTGCCA; Pol-NG S 5ʹ-TGGCAGCGACCCCTCGTCACAATAAAGATGGTGAGCAAGGGCGAGGAGGATAACATG, NG XbaI 5ʹ-CTCCTCTCTAGACTACTTGTACAGCTCGTCCATGCCCAT).

Mutagenesis of this construct was accomplished using the same primers as above.

### Viral stock production

293T cells were seeded at 6 × 10^6^ cells per 10 cm dish and transfected the next day using polyethylenimine. 8 h post transfection, cells were placed in fresh medium. For generation of full-length virus, 293T cells were transfected with 15 μg of proviral plasmid. For generation of imaging constructs, 293T cells were transfected with 6 μg of proviral plasmid, 6 μg of SYN-GP, and 1.2 μg of VSV-G. At 48 h post transfection, supernatants were harvest and passed through a 0.22 μM filter. Titer of full-length infectious viruses was determined by serial dilution on MT4 cells. At 48 h post infection, cells were fixed with 4% PFA and assessed via flow cytometry. Titer of imaging constructs was determined by serial dilution on TZM-bl cells. 48 h post infection, cells were fixed with 0.5% glutaraldehyde and stained with X-gal to visualize number of infected foci.

### Generation of IPMK KO cell lines

The IPMK-targeting guides g1: ATGTACGGGAAGGACAAAGT; g2: GGTGGACTCGATCGCCGGTG; or g3: CCGGCCACCTGATGCGAGAG were designed using the Broad Institute GPP Web Portal and cloned into lentiCRISPRv2 bearing a Hygromycin resistance cassette digested with BsmBI. lentiCRISPR v2 was a gift from Feng Zhang (Addgene plasmid # 52961; http://n2t.net/addgene:52961; RRID:Addgene_52961). VLPs were prepared as above, with the exception that 1 × 10^6^ 293Ts/well were seeded in a 6 well plate and transfected the next day with 1 µg lentiCRISPRv2, 1 µg of Gag-Pol, and 0.2 μg of VSV-G. At 48 h post transduction of target cells with lentiCRISPRv2, cells were placed in selection with 100 μg/mL Hygromycin for ~ 10–14 days. Single cell clones were obtained by limiting dilution, and editing was verified by amplifying and sequencing target loci using primers: IPMK Seq F: 5ʹ-CGCTTCTGCTCTCCGTTATG and IPMK Seq R: 5ʹ-GGATTTGGCGTGCACACCAG and assessment using Synthego ICE, which identifies Indel frequency in Sanger sequencing data (Synthego Performance Analysis, ICE Analysis. 2019. v2.0.). Control cells were obtained similarly, using a lentiCRISPRv2 plasmid not harboring a sgRNA cassette.

### Single cycle infectivity assays

WT control or IPMK KO 293T cells were seeded at 2.5 × 10^5^ cells/well in a 24 well plate and transfected with 625 ng of HIV-1_WT,_ HIV-1_K359A_, or HIV-1_K359A/T371I_ proviral plamids. Virions were prepared as above and titrated on MT4 cells. 24 h post infection, Dextran Sulfate was added (50 µg/mL) to limit infection to a single round. 48 h post infection, cells were fixed with 4% PFA and assessed via flow cytometry.

### Spreading assays

5 × 10^4^ cells per well were seeded in a 96 well plate and infected at an MOI of 0.001. 16 h post infection, cells were washed three times and placed in 5 µM BVM or DMSO control. Supernatants were collected at indicated timepoints, and levels of reverse transcriptase were quantified using the SYBR-PERT assay as previously described [26].

### Western blotting

293Ts were seeded 5 × 10^5^ cells/well in a 12 well dish and transfected the next day with 1.25 µg proviral plasmid. 48 h post transfection, cell lysates and virions pelleted through 20% sucrose (14,000xg for 90 min at 4 °C) were separated on a NuPage 4–12% Bis–Tris Gel (Invitrogen) and subsequently blotted onto a nitrocellulose membrane. Blots were blocked with Intercept Blocking Buffer (Li-Cor) and probed with primary antibody along with a corresponding IRDye 800CW- or IRDye 680-conjugated secondary antibody. Images were acquired using an Odyssey scanner (Li-Cor Biosciences). HIV-1 CA was detected using a human monoclonal anti-p24 (NIH AIDS Reagent Catalog #530).

### Imaging

5 × 10^4^ TZM-bl cells per well were plated in a Lab-Tek Chamber Slide and infected the following day with indicated imaging construct at an MOI of 1. For fixed samples, cells infected in the presence or absence of 5 µM BVM were fixed 48 h post infection and imaged on a DeltaVision OMX SR imaging system using a 60X Widefield oil immersion objective (Olympus) with an exposure time of 50 ms, 10% Transmission, A488 nm laser. For live-cell samples, image acquisition began 26 h post infection, with cells placed in the presence or absence of 5 µM BVM at the time of image acquisition. Images were acquired at 37 °C, 5% CO_2_ at indicated timepoints using a 60X Widefield oil immersion objective with an exposure time of 45 ms, 5% Transmission A488 nm laser. For TIR-FM, cells were imaged approximately 28–30 h post infection in the presence or absence of 5 µM BVM at 37 °C, 5% CO_2_. Images were acquired every 1 min for 90 min using a 60X RING-TIR-FM objective (Olympus Apo N 60X 1.49 Oil) with an exposure time of 100 ms, 10% Transmission A488 nm laser. Representative images were acquired, and all images were analyzed using Fiji (https://fiji.sc/). Briefly, images were auto thresholded and Gag-NG punctae quantified using the Analyze Particles function in Fiji. Reported is the mean ± SD for displayed images (n = 2 per condition).

### Graphing and statistical analysis

All graphs and corresponding statistical analyses were produced and analyzed with Graphpad Prism.

## Supplementary Information

**Additional file 1. **HIV-1 NG_P289S_ live cell widefield microscopy. TZM cells infected with HIV-1 NG_P289S_ and imaged 26 h post infection in the absence of 5 µM BVM. Image acquisition began immediately after BVM addition. Images were acquired every 30 min. Movie labels: Hours:Minutes:Seconds post BVM addition.

**Additional file 2. **HIV-1 NG_P289S_ live cell widefield microscopy. TZM cells infected with HIV-1 NG_P289S_ and imaged 26 h post infection in the absence of 5 µM BVM. Image acquisition began immediately after BVM addition. Images were acquired every 30 min. Movie labels: Hours:Minutes:Seconds post BVM addition.

**Additional file 3. **HIV-1 NG_P289S_ live cell widefield microscopy. TZM cells infected with HIV-1 NG_P289S_ and imaged 26 h post infection in the presence of 5 µM BVM. Image acquisition began immediately after BVM addition. Images were acquired every 30 min. Movie labels: Hours:Minutes:Seconds post BVM addition.

**Additional file 4. **HIV-1 NG_P289S_ live cell widefield microscopy. TZM cells infected with HIV-1 NG_P289S_ and imaged 26 h post infection in the presence of 5 µM BVM. Image acquisition began immediately after BVM addition. Images were acquired every 30 min. Movie labels: Hours:Minutes:Seconds post BVM addition.

**Additional file 5. **HIV-1 NG_P289S_ live cell widefield microscopy at increased time resolution. TZM cells infected with HIV-1 NG_P289S_ and imaged 26 h post infection in the presence of 5 µM BVM. Image acquisition began immediately after BVM addition. Images were acquired every 3 min. Movie labels: Hours:Minutes:Seconds post BVM addition.

**Additional file 6. **HIV-1 NG_P289S_ live cell widefield microscopy at increased time resolution. TZM cells infected with HIV-1 NG_P289S_ and imaged 26 h post infection in the presence of 5 µM BVM. Image acquisition began immediately after BVM addition. Images were acquired every 3 min. Movie labels: Hours:Minutes:Seconds post BVM addition.

**Additional file 7. **HIV-1 NG_P289S_ RING-TIR-FM. TZM cells infected with HIV-1_P289S_ NG and imaged 28 h post infection in the absence of 5 µM BVM using RING-TIRM fluorescence microscopy. Image acquisition began immediately after BVM addition and images were acquired every 60 s. Image labels: Hours:Minutes:Seconds post BVM addition.

**Additional file 8. **HIV-1 NG_P289S_ RING-TIR-FM. TZM cells infected with HIV-1_P289S_ NG and imaged 28 h post infection in the absence of 5 µM BVM using RING-TIRM fluorescence microscopy. Image acquisition began immediately after BVM addition and images were acquired every 60 s. Image labels: Hours:Minutes:Seconds post BVM addition.

**Additional file 9. **HIV-1 NG_P289S_ RING-TIR-FM. TZM cells infected with HIV-1_P289S_ NG and imaged 28 h post infection in the presence of 5 µM BVM using RING-TIRM fluorescence microscopy. Image acquisition began immediately after BVM addition and images were acquired every 60 s. Image labels: Hours:Minutes:Seconds post BVM addition.

**Additional file 10. **HIV-1 NG_P289S_ RING-TIR-FM. TZM cells infected with HIV-1_P289S_ NG and imaged 28 h post infection in the presence of 5 µM BVM using RING-TIRM fluorescence microscopy. Image acquisition began immediately after BVM addition and images were acquired every 60 s. Image labels: Hours:Minutes:Seconds post BVM addition.

## Data Availability

The data and reagents used in this study are available from the corresponding author based on reasonable request.

## Abbreviations

BVM: Bevirimat
CA: Capsid protein
IP_6_: Inositol hexaphosphate
MA: Matrix
MI: Maturation inhibitor
NC: Nucleocapsid
SP1: Spacer peptide 1
SP2: Spacer peptide 2
